# Clinical Impact and Cost-Effectiveness of Expanded Voluntary HIV Testing in India

**DOI:** 10.1371/journal.pone.0064604

**Published:** 2013-05-31

**Authors:** Kartik K. Venkatesh, Jessica E. Becker, Nagalingeswaran Kumarasamy, Yoriko M. Nakamura, Kenneth H. Mayer, Elena Losina, Soumya Swaminathan, Timothy P. Flanigan, Rochelle P. Walensky, Kenneth A. Freedberg

**Affiliations:** 1 Divisions of Infectious Disease, Department of Medicine, Alpert Medical School, Brown University/Miriam Hospital, Providence, Rhode Island, United States of America; 2 Yale School of Medicine, New Haven, Connecticut, United States of America; 3 Y. R. Gaitonde Centre for AIDS Research and Education, Chennai, India; 4 Fenway Health, Boston, Massachusetts, United States of America; 5 Department of Medicine, Beth Israel Deaconess Medical Center, Harvard Medical School, Boston, Massachusetts, United States of America; 6 Department of Clinical Research, Tuberculosis Research Centre, Indian Council of Medical Research, Chennai, India; 7 World Health Organization, Geneva, Switzerland; 8 Divisions of Infectious Diseases, Massachusetts General Hospital, Boston, Massachusetts, United States of America; 9 General Medicine, Massachusetts General Hospital, Boston, Massachusetts, United States of America; 10 Medical Practice Evaluation Center, Department of Medicine, Massachusetts General Hospital, Boston, Massachusetts, United States of America; 11 Divisions of Infectious Disease, Brigham and Women’s Hospital, Boston, Massachusetts, United States of America; 12 Department of Orthopedic Surgery, Brigham and Women’s Hospital, Boston, Massachusetts, United States of America; 13 Harvard University Center for AIDS Research, Harvard Medical School, Boston, Massachusetts, United States of America; 14 Department of Health Policy and Management, Harvard School of Public Health, Boston, Massachusetts, United States of America; 15 Department of Epidemiology, Boston University School of Public Health, Boston, Massachusetts, United States of America; 16 Department of Biostatistics, Boston University School of Public Health, Boston, Massachusetts, United States of America; University of Alabama at Birmingham, United States of America

## Abstract

**Background:**

Despite expanding access to antiretroviral therapy (ART), most of the estimated 2.3 to 2.5 million HIV-infected individuals in India remain undiagnosed. The questions of whom to test for HIV and at what frequency remain unclear.

**Methods:**

We used a simulation model of HIV testing and treatment to examine alternative HIV screening strategies: 1) current practice, 2) one-time, 3) every five years, and 4) annually; and we applied these strategies to three population scenarios: 1) the general Indian population (“national population”), i.e. base case (HIV prevalence 0.29%; incidence 0.032/100 person-years [PY]); 2) high-prevalence districts (HIV prevalence 0.8%; incidence 0.088/100 PY), and 3) high-risk groups (HIV prevalence 5.0%; incidence 0.552/100 PY). Cohort characteristics reflected Indians reporting for HIV testing, with a median age of 35 years, 66% men, and a mean CD4 count of 305 cells/µl. The cost of a rapid HIV test was $3.33. Outcomes included life expectancy, HIV-related direct medical costs, incremental cost-effectiveness ratios (ICERs), and secondary transmission benefits. The threshold for “cost-effective” was defined as 3x the annual *per capita* GDP of India ($3,900/year of life saved [YLS]), or for “very cost-effective” was <1x the annual *per capita* GDP ($1,300/YLS).

**Results:**

Compared to current practice, one-time screening was very cost-effective in the national population (ICER: $1,100/YLS), high-prevalence districts (ICER: $800/YLS), and high-risk groups (ICER: $800/YLS). Screening every five years in the national population (ICER: $1,900/YLS) and annual screening in high-prevalence districts (ICER: $1,900/YLS) and high-risk groups (ICER: $1,800/YLS) were also cost-effective. Results were most sensitive to costs of care and linkage-to-care.

**Conclusions:**

In India, voluntary HIV screening of the national population every five years offers substantial clinical benefit and is cost-effective. Annual screening is cost-effective among high-risk groups and in high-prevalence districts nationally. Routine HIV screening in India should be implemented.

## Introduction

India is home to one of the largest HIV epidemics in the world with an estimated 2.3 to 2.5 million infected individuals [1]. The national HIV prevalence is estimated to be 0.29%, though selected regions and risk groups bear a substantially higher HIV burden [2], [3], [4], [5]. High prevalence regions include most southern Indian states (>0.7%), while low prevalence regions include most northern Indian states (<0.1%). Even within a high prevalence state, such as Andhra Pradesh, multiple districts have an HIV prevalence greater than 20% [1], [6]. National surveillance data also demonstrate elevated HIV prevalence among high-risk groups, including 8.7% among injection drug users (IDU), 5.7% among men who have sex with men (MSM), and 5.4% among female sex workers (FSW) [2].

Despite expanding access to antiretroviral therapy (ART) in India, the majority of those infected are unaware of their HIV status and hence unable to access lifesaving treatment [6], [7], [8], [9]. Given the heterogeneity of the Indian HIV epidemic, current screening guidelines developed by India’s National AIDS Control Organization (NACO) emphasize increased HIV testing among population subgroups identified as being at high risk for HIV infection, specifically IDU, MSM, FSW, and migrants [10]. HIV testing services have also expanded for the general population, such that in the past five years, the number of public-sector HIV voluntary counseling and testing (HIV-VCT) centers has nearly doubled, to 5,135 sites across the country [1], [11], with the goal to test 22 million Indians by 2012 [10]. The expansion of HIV testing is occurring in consort with increasing government-funded access to ART and linkage-to-care programs [1]. Our objective was to assess the clinical impact, cost, and cost-effectiveness of alternative HIV screening strategies in India to provide decision makers with an assessment of the implications of an expanded HIV screening program.

## Methods

### Analytic Overview

We used the Cost-Effectiveness of Preventing AIDS Complications (CEPAC)-International model, a state-transition simulation model of HIV detection and disease in resource-limited settings, to project CD4 count at the time of HIV diagnosis, life expectancy, HIV transmissions, cost, and incremental cost-effectiveness of alternative HIV screening strategies in India. Details about the model structure have been published elsewhere [12], [13], [14], [15], [16], [17], [18]. Input parameters for the model included HIV prevalence and incidence, test acceptance, linkage-to-care, HIV natural history in the absence of treatment, treatment efficacy, and costs of HIV testing, monitoring, and routine care. All data were from India, when available. Life expectancy and costs were discounted at 3% per year [19]. Sensitivity analyses examined uncertainties in model parameters.

To determine the cost-effectiveness of each HIV screening strategy, we adapted the general recommendations of the World Health Organization (WHO) Commission on Macroeconomics and Health, which categorize incremental cost-effectiveness ratios (ICERs) <3x the annual *per capita* Gross Domestic Product (GDP) of a given country as “cost-effective”, and screening strategies with ICERs <1x the *per capita* GDP of the country as “very cost-effective” [20], [21]. India’s *per capita* GDP in 2010 was $1,300 [20], which translates to a threshold for “cost-effective” of <$3,900/year of life saved (YLS).

### HIV Screening Strategies and Settings

We focused on the impact of HIV screening on the general Indian population (i.e. base case), but also assessed two scenarios targeting settings and groups at higher risk for HIV infection: high-prevalence districts and high-risk groups. These three screening scenarios with different underlying HIV prevalence, incidence, and annual rate of “background” HIV testing (i.e. current testing per year in the population, without an expanded screening program) are concordant with classification schemes employed by NACO: 1) national population (0.29% prevalence; 0.032/100PY incidence; 3.2% annual background screening); 2) high prevalence districts (0.8% prevalence; 0.088/100PY incidence; 3.3% annual background screening); and 3) high-risk groups, including MSM, FSW, IDU, migrants, and STD clinic attendees (5.0% estimated aggregate prevalence; 0.552/100 PY incidence; 50% annual background screening) (Table 1) [22], [23]. The comparison screening strategy in each scenario was the current screening frequency in the respective population (i.e. current practice), derived from the proportion of the population that reports having had an HIV test in the past year (background screening), as well as the proportion identified upon presentation with an AIDS-defining OI [1]. For each scenario, we compared three additional HIV screening strategies to current practice: one-time, every five years, and annual screening. HIV-related cost and life expectancy are reported both for the HIV-infected population and for the entire population.

**Table 1:** Screening and disease model input parameters.

Variable	Base case value	Range used insensitivity analyses	Reference
**Baseline cohort characteristics**			
Age, mean years +/− SD at presentation	35+/−5		[30]
Male sex (%)	66		[30]
**HIV prevalence (%)**			
National population	0.29	0.15–0.44	[23]
High prevalence district	0.80	0.40–1.20	[22], [40]
High-risk group	5.00	2.50–7.50	[23]
**Annual incidence/100 person-years**			Model derivation
National population	0.032	0.016–0.048	
High prevalence district	0.088	0.044–0.133	
High-risk group	0.552	0.276–0.829	
**Distribution of initial CD4 count at model initiation,** **mean cells/µl (SD)**			
Acute, primary HIV infection	553 (230)		[58]
Chronic, HIV infection	305 (270)	50–350	[30], [34]
**HIV RNA distribution (%)**			[34]
>100,000 copies/ml	41		
30,001–100,000 copies/ml	26		
10,001–30,000 copies/ml	16		
3,001–10,000 copies/ml	11		
501–3,000 copies/ml	3		
<500 copies/ml	4		
**HIV testing protocols**			
**Rate of background HIV testing, % per year**			[1]
National population	3.2	0–6.4	
High prevalence district	3.3	0–6.6	
High-risk group	50	0–100	
**Sensitivity (%)**	99.6		[10]
**Specificity (%)**	98.0		[10]
**Test acceptance rate (%)**	82	10–100	[40]
**Linkage-to-care rate (%)**	50	10–100	[43]
**Loss-to follow-up (rate/100 PY)** ≤1 year of ART initiation >1year after ART initiation	11.75.8		[30]
**Natural history of HIV disease**			
**Mean monthly CD4 cell decline by HIV RNA level, cells/µl (SD)**			[24]
>30,001 copies/ml	6 (0.255)		
10,001–30,000 copies/ml	5 (0.221)		
3,001–10,000 copies/ml	5 (0.191)		
501–3,000 copies/ml	4 (0.242)		
<500 copies/ml	3 (0.251)		
**Percent monthly risk of severe opportunistic infections** a			[34]
Bacterial	0.0004–0.0022		
Tuberculosis	0.0023–0.0597		
WHO Stage 3–4 visceral^b^	0.0012–0.0338		
WHO Stage 3–4 mucocutaneous^c^	0.0027–0.0478		
Other WHO Stage IV definingIllnesses	0.001–10.0229		
Other severe infections	0.0023–0.0265		
**Percent monthly risk of mild opportunistic infections**			[34]
Bacterial	0.0022–0.0050		
Fungal	0.0032–0.0812		
Other	0.0056–0.0271		
**Efficacy of co-trimoxazole (% reduction in probability of infection)**			[15]
Severe bacterial	49.81		
Mild fungal infections	−46.37^d^		
Stage 3–4 visceral^b^	17.86		
Mild bacterial	48.79		
Other WHO Stage IV definingIllnesses	17.88		
Malaria	88.42		
**Efficacy of ART (% patients with HIV RNA suppression at 24 weeks)**			
First line (NNRTI +2 NRTIs)	73	63–83	[35]
Second line (PI +2 recycled NRTIs)	73	63–83	Assumption
**Discount rate (%)**	3	0–3	[19]
**Costs**			
**HIV testing and care costs ($, USD 2010)**			
Rapid HIV test, including Confirmatory test	3.33	0.5–2x base case	[42]
Co-trimoxazole prophylaxis, Monthly	0.33	0.5–2x base case	[18]
First-line ART, monthly	8.61	0.5–2x base case	[59]
Second-line ART, monthly	55.12	0.5–2x base case	[59]
Minor ART toxicity on first-line, monthly^e^	14.76		[60]
Major drug toxicity on first-line, monthly^e^	160.64		[60]
Routine care	7.23–24.74	0.5–2x base case	[36]
Inpatient hospital care, per day	48.45	0.5–2x base case	[36]
Outpatient hospital care, per visit	16.59	0.5–2x base case	[36]
Acute OI event	24.89–175.77	0.5–2x base case	[36]
CD4 test	6.54	0.5–2x base case	[61]
HIV RNA test	47.96	0.5–2x base case	[61]
**Non-HIV care costs ($)**			
**Overall health expenditure, monthly**	2.88	0.5–2x base case	[39]
**Rate of HIV transmission according to plasma viral load, per 100 person-years**			[45]
<500 copies/ml	0.16		
500–3499 copies/ml	2.06		
3500–9999 copies/ml	4.17		
10000–49999 copies/ml	8.12		
≥50000 copies/ml	9.03		

ART – antiretroviral therapy; WHO – World Health Organization; NNRTI – non-nucleotide reverse transcriptase inhibitor; NRTI – nucleoside reverse transcriptase inhibitor; PI – protease inhibitor; OI – opportunistic infection; SD – standard deviation; PY – person-years; YLS – year of life saved.

a Range due to variation in probability of opportunistic infection acquisition depending on CD4 count.

b Visceral opportunistic infections include: Cryptococcal meningitis, PCP, toxoplasmosis, cryptosporidial diarrhea, parasitic diarrhea, encephalitis, CMV retinitis, non-Hodgkins lymphoma, end stage renal disease, cancer of the vulva, Kaposi’s sarcoma, malignancy, and progressive multifocal leukoencephalopathy.

c Mucocutaneous opportunistic infections include: esophagitis, esophageal candidiasis, oral hairy leukoplakia, and herpes simplex.

d Negative value reflects increased risk of developing mild fungal infections when taking co-trimoxazole.

e See Text S1 for discussion of ART toxicity.

### Disease Model

The CEPAC-International (CEPAC-I) model is a state-transition Monte Carlo simulation model of HIV disease and treatment [14]. Each HIV-infected individual is followed from model entry until death. The natural history of HIV disease is determined by CD4 count decline, which depends on HIV RNA level [24]. HIV morbidity (i.e. opportunistic infections [OIs]) and mortality are CD4 count-dependent, with higher morbidity and mortality at lower CD4 counts [25]. ART reduces HIV RNA levels, increases CD4 counts, and decreases HIV-related morbidity and mortality [26]. ART regimens follow guidelines from NACO and the WHO [27], [28], [29]. ART-eligible individuals receive a first-line non-nucleoside reverse transcriptase inhibitor (NNRTI)-based regimen followed by a second-line regimen using a boosted protease inhibitor (PI), if needed. HIV-infected individuals in care are assumed to have CD4 count tests performed every 6 months; are treated with co-trimoxazole prophylaxis at CD4 counts ≤200 cells/µl; receive the first of two sequential ART regimens once their CD4 counts fall to <350 cells/µl or after they develop a WHO Stage III–IV disease; and are treated for any acute OIs that develop [29]. In the absence of HIV RNA monitoring, detection of treatment failure, based on observation of a 50% CD4 decline from peak, a CD4 drop below pre-ART nadir, a CD4 count <100 cells/µl, or a severe OI while receiving ART, triggers a switch to the second (and final) available ART regimen [27]. HIV-infected individuals who are tested and linked-to-care, but not yet eligible for ART, are assumed to be monitored with clinic visits every 3 months and CD4 counts every 6 months until their CD4 counts are <350 cells/µl. Once in care, HIV-infected individuals can be lost to follow-up at a frequency consistent with observational data from India, with the possibility of subsequently returning to care if lost [30].

### Screening Model

Entry into the disease model is determined by a population-level screening model that includes HIV prevalence and incidence. Further details about the Screening model can be found in published reports [31], [32]. Briefly, this model allows the user to define cohort characteristics (e.g. distributions of age, sex, and, for HIV-infected individuals, CD4 count, HIV RNA, and history of OI). Given the demographic characteristics of HIV-uninfected individuals and the user-defined incidence of HIV infection, the model determines the number of incident HIV cases in the simulation. In the model, individuals are offered an HIV test at a specified screening frequency. Those who accept testing, based on a user-defined probability of test acceptance, receive one rapid HIV test; a reactive test triggers a confirmatory rapid test.

Individuals with undetected HIV infection in the Screening model can be diagnosed via one of three mechanisms: 1) background testing, which occurs at VCT sites, tuberculosis clinics, STD centers, or antenatal clinics, based on current testing patterns; 2) presentation with an AIDS-defining OI; or 3) an expanded HIV screening program if available, as described in this analysis. We define “current practice” as detection via mechanisms 1 or 2. The analysis involves a conservative approach towards expanded screening in which it is assumed that HIV detection by “current practice” leads to successful linkage-to-care. However, in the expanded screening programs, the rates of test acceptance, test sensitivity and specificity, and linkage-to-care are user-specified. Only those who are tested for HIV, diagnosed, linked-to-care, and meet eligibility criteria receive ART and OI prophylaxis and accrue HIV-related costs of care. Those not yet ART-eligible accrue only monitoring costs and non-HIV care costs.

### Disease Model Input Parameters

#### Cohort characteristics and disease progression

Baseline cohort characteristics are from the national government HIV testing program and the Y.R. Gaitonde Centre for AIDS Research and Education (YRG CARE), a community-based tertiary care facility in South India (Table 1) [30], [33]. The mean age at Screening model entry is 35 (SD±5) years [30]. Individuals with undiagnosed prevalent HIV infection have a CD4 count and HIV RNA distribution as reported for Indian cohorts [30], [34]; the mean CD4 count of chronically HIV-infected individuals in the cohort is 305 cell/µl (SD 270 cell/µl) [34], which is concordant with internal calibration by the CEPAC-I model (See Text S1).

Data on HIV natural history and prophylaxis efficacy have been published elsewhere [15], [24]. The efficacy of first-line ART, defined as HIV RNA suppression (<50 copies/ml) at 48weeks, is 73% [35]. Due to the absence of data on second-line ART efficacy specifically from India, but consistent with the literature, we utilize the same efficacy for second-line ART as for first-line ART [35]. Additional data on clinical inputs have been published in previous India-based analyses [12], [13], [18]. For those enrolled in HIV care, the rate of loss to follow-up from treatment is 11.7/100 PY for the first 12 months on treatment and 5.8/100 PY thereafter [30]. Individuals lost to follow-up have a 50% probability of returning to HIV care upon developing a WHO Stage III–IV OI or tuberculosis.

#### Resource utilization and costs of care

Costs associated with routine HIV care, acute HIV-associated OIs, and death are derived using resource utilization data from the YRG CARE observational database and a unit cost analysis [34], [36]. ART drug costs are from NACO [37]. Monthly per-person costs of first- and second-line ART are $8.61 and $55.12, and the monthly cost of co-trimoxazole prophylaxis is $0.33 [37], [38]. Cost for an outpatient visit is $16.59, and for an inpatient day $48.45 [36]. Non-HIV care costs are applied monthly to both HIV-uninfected and HIV-infected individuals, and are estimated at $2.88 per month, which is the overall mean monthly health expenditure of Indian citizens as estimated from population-level WHO data [39]. All costs are standardized to 2010 US dollars using India’s GDP deflator [20].

### Screening Model Input Parameters

#### HIV prevalence and incidence

We utilize HIV prevalence data from NACO and the National Family Health Survey [23], [40]. Due to lack of available HIV incidence data, we calculate annual incidence estimates from the prevalence and model-derived duration of infection (Text S1) [22], [23], [40].

#### HIV testing and outcomes

Based on data from the Indian national testing program, the annual background testing rate is estimated at 3.2% per year for the general population, but varies by district and group [1], [22], [23], [40]. Employing these estimates for the national background testing rate and data from YRGCARE on the mean CD4 count of ART-naïve patients, we calibrate the percentage of severe OIs (i.e. WHO Stage III and IV diseases and tuberculosis) that trigger clinical detection of HIV to be 10% [1], [34], which is consistent with previous Indian estimates [18]. We use a point-of-care HIV test (99.6% sensitivity and 98.0% specificity), based on earlier Indian studies [10], [41]. Reactive results are confirmed by a second rapid test [10], [42]. Test acceptance (82%) is based on earlier Indian population-based survey data [40]; and linkage-to-care frequency (50%) is based on HIV testing at Indian TB clinics [40], [43]. The cost of a rapid test is $3.33, including both the confirmatory test for positives and the salary of the counselor [42]. This cost is varied over a range of values in sensitivity analysis (Text S1).

### Secondary Transmission

Recognizing that expanded HIV screening and treatment at the population level may have transmission benefits in addition to individual clinical benefits, we project the number of secondary incident HIV cases over six years (as a reasonable time span to evaluate the public health impact given testing every 5 years was being assessed as an intervention) [31], [44]. HIV transmission rates according to HIV RNA level, which vary from 0.16 per 100 PY with HIV RNA <400 copies/ml to 9.03 per 100 PY with HIV RNA ≥50,000 copies/ml, are from a recent meta-analysis [45]. These rates are consistent with data on the impact of ART on preventing HIV infection [46], [47]. To estimate the number of secondary HIV cases, we aggregate the number of person-years spent by treatment-naïve individuals at each HIV RNA level and multiply by the corresponding HIV transmission rate. We then sum the resulting number of transmissions at each HIV RNA level to generate an overall estimate of secondary HIV transmission for each screening strategy.

### Sensitivity Analysis

We perform extensive univariate and multivariate sensitivity analyses for each of the three population screening scenarios by varying parameter values for HIV prevalence and incidence, background HIV testing rates, test acceptance, linkage-to-care, treatment efficacy and availability, and the costs of testing, treatment, and care.

## Results

### National Population

#### Base case analysis

The mean CD4 count at diagnosis in the national population “current practice” HIV screening strategy was 201 cells/µl for prevalent cases and 314 cells/µl for incident cases. This increased to 290 cells/µl for prevalent cases with one-time testing (no increase for incident cases) and to 312 cells/µl for prevalent cases and 464 cells/µl for incident cases with annual testing. The discounted life expectancy of HIV-infected individuals from time of entry into the screening model was 184.2 months (15.4 years; undiscounted 285.1 months, 23.8 years), and the discounted life expectancy in the overall population was 253.6 months (21.1 years; undiscounted 432.9 months, 36.1 years; Table 2). The addition of a one-time HIV screen at a mean age of 35 years increased discounted life expectancy to 188.5 months (undiscounted 291.0 months) for HIV-infected individuals; for the overall population, the discounted life expectancy increased to 253.7 months (undiscounted 433.0 months). Screening every five years or annually increased the discounted life expectancy of HIV-infected individuals to 196.1 and 208.2 months (undiscounted 307.6 and 331.6 months). As the HIV testing frequency increased, more people were detected with HIV through screening programs, in contrast to background screening or presentation with an opportunistic infection (Table 2).

**Table 2:** Base case results for an analysis of HIV screening in India.

	HIV testing frequency			
	Current practice	One-time	Every 5 years	Annually
**National population**				
***HIV-infected population***				
*Mean CD4 count at detection (cells/µl)*				
Prevalent cases	201	290	289	312
Incident cases	314	314	383	464
Undiscounted per person life expectancy (months) a	285.1	291.0	307.6	331.6
Discounted per person life expectancy (months)^a^	184.2	188.5	196.1	208.2
Discounted per person costs ($)	1,137	1,385	1,843	2,597
*Mechanism of HIV Detection (%)*				
Background screening	14	13	10	5
Presentation with opportunistic infection	14	14	10	4
Screening Program	0	7	34	74
Never detected	72	66	46	17
***Overall population***				
Undiscounted per person life expectancy (months)	432.9	433.0	433.2	433.6
Discounted per person life expectancy (months)	253.6	253.7	253.8	253.9
Discounted per person costs ($)	739	745	762	818
***Cost-effectiveness ratio ($/YLS)***	–	1,100	1,900	4,000
	**HIV testing frequency**			
	**Current practice**	**One-time**	**Every 5 years**	**Annually**
**High prevalence district**				
***HIV-infected population***				
*Mean CD4 count at detection (cells/µl)*				
Prevalent cases	202	290	289	312
Incident cases	316	316	383	464
Undiscounted per person life expectancy (months) a	283.8	289.8	306.3	330.2
Discounted per person life expectancy (months)^a^	183.5	187.9	195.5	207.6
Discounted per person costs ($)	1,145	1,396	1,843	2,607
*Mechanism of HIV Detection (%)*				
Background screening	14	13	10	5
Presentation with opportunistic infection	15	14	10	4
Screening Program	0	7	34	74
Never detected	71	66	46	17
***Overall population***				
Undiscounted per person life expectancy (months)	427.9	428.2	428.8	429.7
Discounted per person life expectancy (months)	251.4	251.5	251.8	252.3
Discounted per person costs ($)	748	760	787	861
***Cost-effectiveness ratio ($/YLS)***	–	800	1,100	1,900
	**HIV testing frequency**			
	**Current practice**	**One-time**	**Every 5 years**	**Annually**
**High-risk group**				
***HIV-infected population***				
*Mean CD4 count at detection (cells/µl)*				
Prevalent cases	306	316	315	321
Incident cases	467	467	475	496
Undiscounted per person life expectancy (months) a	316.2	318.8	320.3	324.5
Discounted per person life expectancy (months)^a^	200.6	202.4	203.2	205.5
Discounted per person costs ($)	2,554	2,669	2,720	2,893
*Mechanism of HIV Detection (%)*				
Background screening	77	72	65	44
Presentation with opportunistic infection	4	4	3	2
Screening Program	0	8	17	44
Never detected	19	17	15	10
***Overall population***				
Undiscounted per person life expectancy (months)	399.8	400.4	400.7	401.6
Discounted per person life expectancy (months)	239.1	239.5	239.7	240.2
Discounted per person costs ($)	1,116	1,143	1,162	1,235
***Cost-effectiveness ratio ($/YLS)***	–	800	1,300	1,800

PY – person-years; YLS – year of life saved.

a Calculated from the time of model entry – includes time to HIV infection (incident cases only) and detection.

Expanded screening in the national population increased the mean discounted per person lifetime cost of care from $739 for current practice to $745, $762, and $818 for one-time, every five years, and annual screening. Compared to current practice, one-time screening had an ICER of $1,100/YLS. Screening every five years resulted in an ICER of $1,900/YLS compared to one-time screening. Screening annually resulted in an ICER of $4,000/YLS compared to screening every five years.

#### Secondary transmission benefits

Under current screening practice in the national population, we calculated over 6.92 secondary HIV transmissions per 100,000 people over a 6-year period (Table 3). A one-time national population screen could avert 2.5% of these secondary transmissions. Screening every five years or annually could avert a further decrease of 1.5% and 6.9%.The number of HIV secondary transmissions could be further reduced by improving test acceptance and linkage-to-care rates, as well as improving viral suppression on ART (Tables S2 and S3).

**Table 3:** Secondary transmission of HIV in the first 6 years following screening program implementation.

	HIV testing frequency			
	Current practice	One-time	Every 5 years	Annually
**National population**				
***Prevalence 0.29%, Incidence 0.032/100PY, background testing 3.2% per year***				
Number of secondary cases (per 100,000)	6.92	6.75	6.65	6.19
Incremental HIV cases averted^a^ (per 100,000)	–	0.17	0.10	0.46
% incremental decrease	–	2.5	1.5	6.9
**High prevalence district**				
***Prevalence 0.8%, Incidence 0.088/100PY, background testing 3.3% per year***				
Number of secondary cases (per 100,000)	6.99	6.82	6.72	6.26
Incremental HIV cases averted^a^ (per 100,000)	–	0.17	0.10	0.46
% incremental decrease	–	2.4	1.5	6.8
**High-risk group**				
***Prevalence 5.0%, Incidence 0.552/100PY, background testing 50% per year***				
Number of secondary cases (per 100,000)	6.84	6.84	6.81	6.70
Incremental HIV cases averted^a^ (per 100,000)	–	0.0	0.03	0.11
% incremental decrease	–	0.0	0.4	1.6

PY – person-years.

Transmission coefficient ranges from 0.16/100 PY to 9.03/100 PY depending on HIV RNA level [45].

a Incremental HIV cases averted relative to those averted with the next less frequent HIV testing strategy.

#### Sensitivity analyses

**Figure 1:**
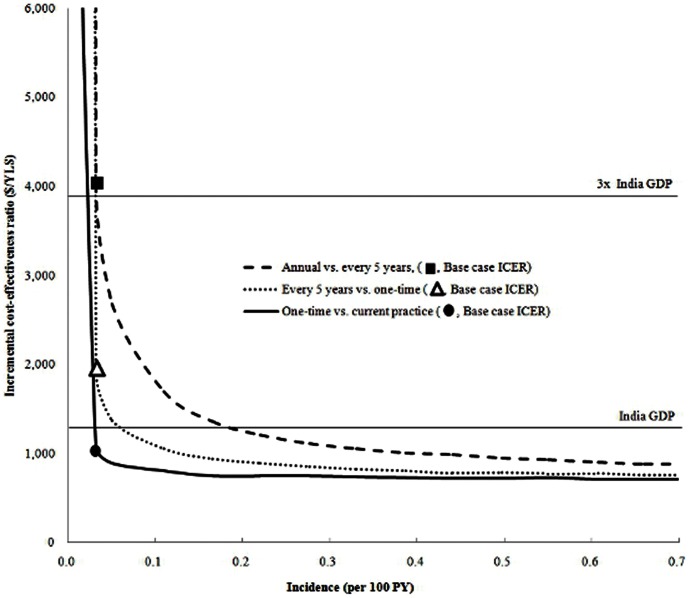
Impact of varying HIV incidence on the incremental cost-effectiveness of various testing frequencies. The incidence (horizontal axis) was increased incrementally from 0 to 0.7/100 PY. The bold line indicates one-time screening compared with the current practice, the dotted line indicates screening every five years compared with one-time screening, and the dashed line indicates annual screening compared with screening every five years.The circle, triangle, and square indicate the base case incremental cost-effectiveness ratio for one-time screening compared with the current practice, screening every five years compared with one-time screening, and annual screening compared with screening every five years. The horizontal lines indicate the threshold values for “very cost-effective” (1x *per capita* India GDP) and “cost-effective” (3x *per capita* India GDP).

While changes in HIV incidence and prevalence had an impact on the ICER for screening, they did not affect the policy conclusions substantially. Decreasing and increasing HIV prevalence by 50% of the base case, with the resulting change in derived incidence, yielded ICERs for national screening every five years of $2,900/YLS and $1,500/YLS, respectively (Table S1). As the incidence of HIV increased, particularly above 0.2/100 PY, the ICER progressively diminished. Only when the incidence dropped below 0.03/100 PY did the ICER for one time screening go above the 3x GDP threshold (Figure 1).

**Figure 2:**
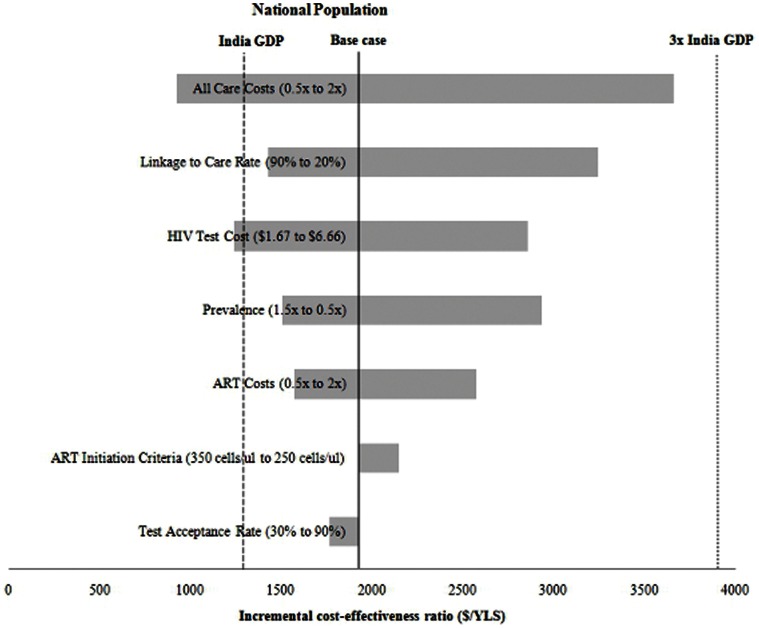
One-way sensitivity analyses: Screening every five years vs. one-time testing in the national population. The width of the horizontal bars represents the difference in the incremental cost-effectiveness ratio ($/year of life saved, YLS) between the range described in parentheses in the figure. The bold line represents the base case incremental cost-effectiveness ratio. The dashed line is the threshold value for “very cost-effective” (1x *per capita* India GDP) and the dotted line is the threshold for “cost-effective” (3x *per capita* India GDP).

In an additional one-way sensitivity analysis, linkage-to-care rates, overall care costs, and HIV test costs had the greatest impact on results (Figure 2). When the costs of ART, CD4 count and HIV RNA monitoring, and non-HIV treatment costs were varied individually, these parameters did not have a major impact on the findings (Table S1).

In a two-way sensitivity analysis, we varied both the test acceptance and linkage-to-care rates from 10% to 90%. Below 20% test acceptance and 20% linkage-to-care, national testing every five years was no longer cost-effective.

### High Prevalence Districts and High-risk Groups

#### Base case analysis

In high prevalence districts, with background testing similar to the national population (3.3% per year), one-time screening yielded an ICER of $800/YLS compared to current practice (Table 2). Screening every five years and annually yielded ICERs of $1,100/YLS and $1,900/YLS.

Among high-risk populations, with a much higher reported background testing rate (50% per year), one-time screening had an ICER of $800/YLS relative to background testing (Table 2). Screening every five years ($1,300/YLS) and annually ($1,800/YLS) were both cost-effective. If the background testing rate was decreased to 25%, one-time ($700/YLS), every five years ($1,000/YLS), and annual screening ($1,200/YLS) became even more attractive.

#### Secondary transmission benefits

We observed a prevention impact of wider HIV screening in high-prevalence districts similar to that in the national population. The proportion of averted cases was lower in high-risk groups compared to high prevalence districts, likely due to a higher frequency of reported background testing, and thus earlier HIV diagnosis even without a screening program implemented (Table 3).

#### Sensitivity analyses

In high prevalence districts and among high-risk groups, decreasing the HIV prevalence by 50% still yielded annual screening results well below the 3x *per capita* GDP cost-effectiveness threshold (Figures S1 and S2). Annual testing added cost with limited additional health benefits compared to testing every five years. The results remained cost-effective when all costs were doubled, even with annual testing.

## Discussion

We modeled the impact of various HIV screening strategies in India and found that screening every five years in the national adult population would increase mean CD4 count at HIV diagnosis, improve survival among the HIV-infected population, modestly reduce secondary HIV infections at six years, and be cost-effective by WHO criteria. We found that annual screening is economically justifiable in specific sub-populations at increased risk for HIV. Due to uncertainty over HIV prevalence and incidence data in India, we varied these estimates widely. Even if HIV prevalence was halved, screening every five years nationally, and annual screening among high-prevalence districts and high-risk groups, was still cost-effective when compared to a threshold of 3x India’s annual *per capita* GDP ($3,900).

Previous studies have identified one-time, routine HIV screening to be cost-effective in many countries with a lower undiagnosed HIV prevalence than in India, such as in the United States and France [31], [48], [49]. In India, a majority of HIV-infected individuals are unaware of their HIV status [1]. Clinical studies have documented that HIV-infected Indians continue to be detected late in the course of their HIV disease, with 85% registering for ART when their CD4 count is already <250 cells/µl [50]. Routine testing could detect HIV infection at an earlier disease stage, link infected individuals to needed care, and decrease the rate of secondary HIV transmission.

The findings of this study were robust across a wide range of sensitivity analyses, including increasing HIV screening costs, as well as treatment and monitoring costs. This suggests that variations in programs by site, clinical services, or other operational differences are unlikely to have a marked impact on the overall findings. It is also likely that the costs of HIV screening and disease management, including CD4 count and HIV RNA monitoring, will continue to decrease based on the wider utilization of new technologies in India [51], [52]. Given limited test acceptance and linkage-to-care following HIV detection, the expansion of HIV screening services will need to occur in consort with interventions aimed at improving both test acceptance and developing better mechanisms of linking HIV-infected individuals to treatment programs. Data from TB testing programs in India that conduct HIV screening suggest that linkage-to-care remains at only 50%; linkage is likely even worse among the Indian general population [43]. In light of nationwide primary prevention programs, such as the Gates Foundation-funded Avahan initiative, which has averted an estimated 100,000 new HIV cases over five years among high-risk groups residing in high prevalence Indian states [53], it is possible that more Indians will seek HIV testing and, for those found to be infected, will be linked-to-care.

When assessing the transmission benefits associated with expanded HIV screening, this analysis suggests that ART-associated reductions in HIV RNA could have an impact, albeit relatively modest, over the short-term on reducing secondary infections. Emerging data from resource-limited settings suggest that accessing VCT is associated with a decrease in unprotected sex [7], [54], [55], [56]. This analysis did not account for the additional prevention benefits associated with expanded testing. Additionally, HIV-infected Indians also report decreased sexual risk behaviors following enrollment in ART programs [57]. Recent clinical trial data support the preventive impact of ART in reducing HIV transmission among serodiscordant couples in resource-limited settings [47]. By not changing the HIV incidence due to decreased sexual risk behaviors over time, we aimed to generate conservative estimates of projected life expectancy, cost-effectiveness, and secondary cases averted of an HIV screening program.

There are several limitations to this analysis. The model combines data from multiple sources to project the long-term benefits of alternative HIV screening strategies. Disease progression parameters were from a tertiary care HIV center in South India, but these clinical inputs were unlikely to be site-dependent; demographic inputs were taken from the Indian government to better reflect the characteristics of a national screening program. Given limited HIV incidence data from India, HIV incidence rates were estimated from back-calculations utilizing available prevalence data [48]. To address the lack of population-level data examining linkage-to-care and test acceptance in India, we conducted sensitivity analyses to test the robustness of the results. With the scale-up of ART, it is unclear whether district-level testing facilities would be able to accommodate the increased patient burden resulting from routine HIV testing. We did not include a start-up cost for test sites that do not yet exist, but the results were robust to test and care costs varied widely in sensitivity analyses. In light of both logistical challenges as well as limited funding, we adopted a conservative approach to provide decision makers with a realistic assessment of the clinical impact and cost-effectiveness of expanded HIV screening in India.

Routine HIV screening every five years is cost-effective in India and should be implemented on a national, population-wide basis to address the growing Indian HIV epidemic. More frequent screening is warranted among Indian sub-populations with higher HIV prevalence and incidence. This increased frequency of testing, combined with the expansion of ART services, and recent efforts towards earlier ART initiation, will improve outcomes in those with HIV disease, decrease HIV transmission, and be cost-effective in India.

## Supporting Information

Figure S1 **One-way sensitivity analyses: Screening every five years vs. one-time testing in high prevalence districts.** The width of the horizontal bars represents the difference in the incremental cost-effectiveness ratio ($/year of life saved, YLS) between the range described in parentheses in the figure. The bold line represents the base case incremental cost-effectiveness ratio. The dashed line is the cut-off value for “very cost-effective” (1x *per capita* India GDP) and the dotted line is the cut-off for “cost-effective” (3x *per capita* India GDP).
(TIF)

Figure S2 **One-way sensitivity analyses: Screening every five years vs. one-time testing in high risk groups.** The width of the horizontal bars represents the difference in the incremental cost-effectiveness ratio ($/year of life saved, YLS) between the range described in parentheses in the figure. The bold line represents the base case incremental cost-effectiveness ratio. The dashed line is the cut-off value for “very cost-effective” (1x *per capita* India GDP) and the dotted line is the cut-off for “cost-effective” (3x *per capita* India GDP).
(TIF)

Table S1 **Sensitivity analysis on testing parameters.** National population.
(DOCX)

Table S2 **Sensitivity analysis on secondary transmission.** 90% linkage-to-care.
(DOCX)

Table S3 **Sensitivity analysis on secondary transmission.** 90% test acceptance.
(DOCX)

Text S1 **Technical appendix.**
(DOCX)
