# Supplementary figures and images for: Clinical Impact and Cost-Effectiveness of Expanded Voluntary HIV Testing in India
Source: PLoS One. 2013 May 31;8(5):e64604. doi: 10.1371/journal.pone.0064604 (PMC3669338; doi:10.1371/journal.pone.0064604)

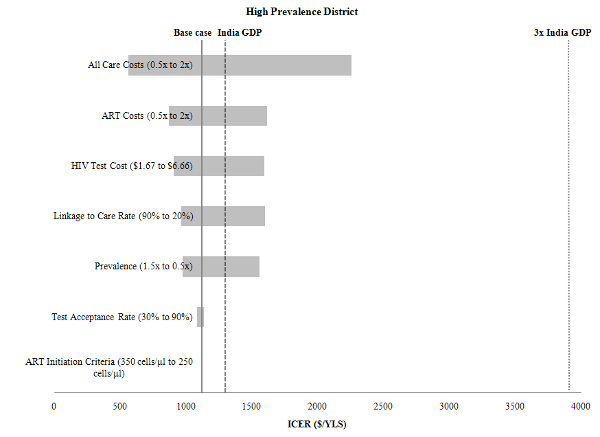

Supplement: Figure S1 — One-way sensitivity analyses: Screening every five years vs. one-time testing in high prevalence districts. The width of the horizontal bars represents the difference in the incremental cost-effectiveness ratio ($/year of life saved, YLS) between the range described in parentheses in the figure. The bold line represents the base case incremental cost-effectiveness ratio. The dashed line is the cut-off value for “very cost-effective” (1x per capita India GDP) and the dotted line is the cut-off for “cost-effective” (3x per capita India GDP). (TIF) [file pone.0064604.s001.tif]

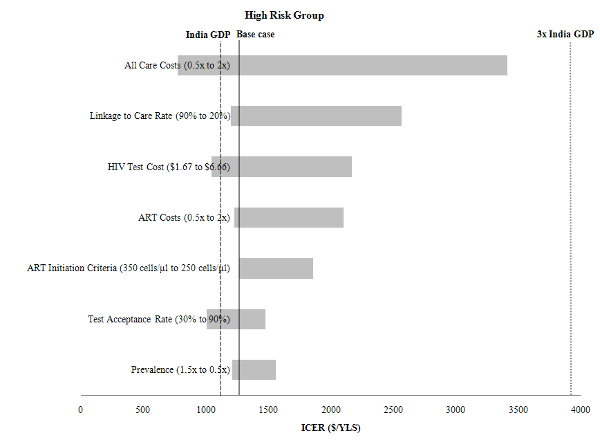

Supplement: Figure S2 — One-way sensitivity analyses: Screening every five years vs. one-time testing in high risk groups. The width of the horizontal bars represents the difference in the incremental cost-effectiveness ratio ($/year of life saved, YLS) between the range described in parentheses in the figure. The bold line represents the base case incremental cost-effectiveness ratio. The dashed line is the cut-off value for “very cost-effective” (1x per capita India GDP) and the dotted line is the cut-off for “cost-effective” (3x per capita India GDP). (TIF) [file pone.0064604.s002.tif]
