# Supplementary material for: Clinical Impact and Cost-Effectiveness of Expanded Voluntary HIV Testing in India
Source: PLoS One. 2013 May 31;8(5):e64604. doi: 10.1371/journal.pone.0064604 (PMC3669338; doi:10.1371/journal.pone.0064604)
Supplement: Table S1 — Sensitivity analysis on testing parameters. National population. (DOCX) [file pone.0064604.s003.docx]

**Table S1. Sensitivity analysis on testing parameters. National population.**

|  | **HIV testing frequency** | | | |
| --- | --- | --- | --- | --- |
|  | **Current practice** | **One-time** | **Every 5 years** | **Annually** |
| **Increasing HIV prevalence by 50%** |  |  |  |  |
| ***HIV-infected population*** |  |  |  |  |
| Discounted per person life expectancy (months)^a^ | 183.8 | 188.2 | 195.8 | 207.9 |
| ***Overall population*** |  |  |  |  |
| Discounted per person life expectancy (months) | 252.9 | 253.0 | 253.2 | 253.5 |
| Discounted per person costs ($) months | 741 | 749 | 769 | 830 |
| ***Cost-effectiveness ratio ($/YLS)*** | --- | 1,000 | 1,500 | 2,900 |
|  |  |  |  |  |
| **Decreasing HIV prevalence by 50%** |  |  |  |  |
| ***HIV-infected population*** |  |  |  |  |
| Discounted per person life expectancy (months)^a^ | 184.3 | 188.7 | 196.2 | 208.3 |
| ***Overall population*** |  |  |  |  |
| Discounted per person life expectancy (months) | 254.2 | 254.3 | 254.3 | 254.4 |
| Discounted per person costs ($) months | 737 | 741 | 754 | 806 |
| ***Cost-effectiveness ratio ($/YLS)*** | **---** | 1,700 | 2,900 | 7,500 |
|  | | | | |

|  | **HIV testing frequency** | | | |
| --- | --- | --- | --- | --- |
|  | **Current practice** | **One-time** | **Every 5 years** | **Annually** |
| **10% Background Testing/ year (Base case = 3.2%/year)** |  |  |  |  |
| ***HIV-infected population*** |  |  |  |  |
| Discounted per person life expectancy (months)^a^ | 191.6 | 195.2 | 200.1 | 209.0 |
| ***Overall population*** |  |  |  |  |
| Discounted per person life expectancy (months) | 253.7 | 253.8 | 253.8 | 253.9 |
| Discounted per person costs ($) months | 745 | 751 | 765 | 819 |
| ***Cost-effectiveness ratio ($/YLS)*** | **---** | 1,400 | 2,300 | 5,500 |
|  |  |  |  |  |
| **80% Background Testing/year (Base case = 3.2%/year)** |  |  |  |  |
| ***HIV-infected population*** |  |  |  |  |
| Discounted per person life expectancy (months)^a^ | 210.0 | 211.2 | 211.5 | 212.5 |
| ***Overall population*** |  |  |  |  |
| Discounted per person life expectancy (months) | 254.0 | 254.0 | 254.0 | 254.0 |
| Discounted per person costs ($) months | 761 | 765 | 775 | 822 |
| ***Cost-effectiveness ratio ($/YLS)*** | **---** | 2,300 | DOM | 46,800 |

|  | **HIV testing frequency** | | | |
| --- | --- | --- | --- | --- |
|  | **Current practice** | **One-time** | **Every 5 years** | **Annually** |
| **30% Test Acceptance (Base case = 82%)** |  |  |  |  |
| ***HIV-infected population*** |  |  |  |  |
| Discounted per person life expectancy (months)^a^ | 184.2 | 185.8 | 188.8 | 198.2 |
| ***Overall population*** |  |  |  |  |
| Discounted per person life expectancy (months) | 253.6 | 253.6 | 253.7 | 253.8 |
| Discounted per person costs ($) months | 739 | 741 | 747 | 772 |
| ***Cost-effectiveness ratio ($/YLS)*** | **---** | 1,300 | 1,800 | 2,300 |
|  |  |  |  |  |
| **90% Test Acceptance (Base case = 82%)** |  |  |  |  |
| ***HIV-infected population*** |  |  |  |  |
| Discounted per person life expectancy (months)^a^ | 184.1 | 188.9 | 197.1 | 209.3 |
| ***Overall population*** |  |  |  |  |
| Discounted per person life expectancy (months) | 253.6 | 253.7 | 253.8 | 253.9 |
| Discounted per person costs ($) months | 739 | 746 | 764 | 824 |
| ***Cost-effectiveness ratio ($/YLS)*** | **---** | 1,200 | 1,900 | 4,200 |

|  | **HIV testing frequency** | | | |
| --- | --- | --- | --- | --- |
|  | **Current practice** | **One-time** | **Every 5 years** | **Annually** |
| **20% Linkage-to-Care (Base case = 50%)** |  |  |  |  |
| ***HIV-infected population*** |  |  |  |  |
| Discounted per person life expectancy (months)^a^ | 184.2 | 185.9 | 189.3 | 199.2 |
| ***Overall population*** |  |  |  |  |
| Discounted per person life expectancy (months) | 253.6 | 253.6 | 253.7 | 253.8 |
| Discounted per person costs ($) months | 739 | 743 | 756 | 810 |
| ***Cost-effectiveness ratio ($/YLS)*** | **---** | 2,400 | 3,200 | 4,700 |
|  |  |  |  |  |
| **90% Linkage-to-Care (Base case = 50%)** |  |  |  |  |
| ***HIV-infected population*** |  |  |  |  |
| Discounted per person life expectancy (months)^a^ | 184.2 | 192,0 | 204.1 | 212.7 |
| ***Overall population*** |  |  |  |  |
| Discounted per person life expectancy (months) | 253.6 | 253.7 | 253.9 | 254.0 |
| Discounted per person costs ($) months | 739 | 748 | 768 | 822 |
| ***Cost-effectiveness ratio ($/YLS)*** | **---** | 1,000 | 1,400 | 5,000 |

|  | **HIV testing frequency** | | | |
| --- | --- | --- | --- | --- |
|  | **Current practice** | **One-time** | **Every 5 years** | **Annually** |
| **0.5x Base Case Test Cost** |  |  |  |  |
| ***HIV-infected population*** |  |  |  |  |
| Discounted per person life expectancy (months)^a^ | 184.2 | 188.5 | 196.1 | 208.2 |
| ***Overall population*** |  |  |  |  |
| Discounted per person life expectancy (months) | 253.6 | 253.7 | 253.8 | 253.9 |
| Discounted per person costs ($) months | 739 | 744 | 755 | 789 |
| ***Cost-effectiveness ratio ($/YLS)*** | **---** | 1,000 | 1,200 | 2,400 |
|  |  |  |  |  |
| **2x Base Case Test Cost** |  |  |  |  |
| ***HIV-infected population*** |  |  |  |  |
| Discounted per person life expectancy (months)^a^ | 184.2 | 188.5 | 196.1 | 208.2 |
| ***Overall population*** |  |  |  |  |
| Discounted per person life expectancy (months) | 253.6 | 253.7 | 253.8 | 253.9 |
| Discounted per person costs ($) months | 739 | 748 | 774 | 877 |
| ***Cost-effectiveness ratio ($/YLS)*** | **---** | 2,000 | 2,900 | 7,300 |

|  | **HIV testing frequency** | | | |
| --- | --- | --- | --- | --- |
|  | **Current practice** | **One-time** | **Every 5 years** | **Annually** |
| **0.5x Base Case 2^nd^ Line ART Cost** |  |  |  |  |
| ***HIV-infected population*** |  |  |  |  |
| Discounted per person life expectancy (months) ^a^ | 184.2 | 188.6 | 196.1 | 208.2 |
| ***Overall population*** |  |  |  |  |
| Discounted per person life expectancy (months) | 253.6 | 253.7 | 253.8 | 253.9 |
| Discounted per person costs ($) months | 736 | 742 | 756 | 809 |
| ***Cost-effectiveness ratio ($/YLS)*** | **---** | 1,100 | 1,600 | 3,900 |
|  |  |  |  |  |
| **0.5x Base Case ART Costs (both lines)** |  |  |  |  |
| ***HIV-infected population*** |  |  |  |  |
| Discounted per person life expectancy (months)^a^ | 184.2 | 188.5 | 196.1 | 208.2 |
| ***Overall population*** |  |  |  |  |
| Discounted per person life expectancy (months) | 253.6 | 253.7 | 253.8 | 253.9 |
| Discounted per person costs ($) months | 736 | 741 | 755 | 808 |
| ***Cost-effectiveness ratio ($/YLS)*** | **---** | 1,100 | 1,600 | 3,700 |

|  | **HIV testing frequency** | | | |
| --- | --- | --- | --- | --- |
|  | **Current practice** | **One-time** | **Every 5 years** | **Annually** |
| **2x Base Case ART Costs (both lines)** |  |  |  |  |
| ***HIV-infected population*** |  |  |  |  |
| Discounted per person life expectancy (months)^a^ | 184.2 | 188.5 | 196.0 | 208.2 |
| ***Overall population*** |  |  |  |  |
| Discounted per person life expectancy (months) | 253.6 | 253.7 | 253.8 | 253.9 |
| Discounted per person costs ($) months | 745 | 753 | 774 | 838 |
| ***Cost-effectiveness ratio ($/YLS)*** | **---** | 1,500 | 2,600 | 4,400 |
|  |  |  |  |  |
| **0.5x Base Case All Costs** |  |  |  |  |
| ***HIV-infected population*** |  |  |  |  |
| Discounted per person life expectancy (months)^a^ | 184.2 | 188.5 | 196.1 | 208.2 |
| ***Overall population*** |  |  |  |  |
| Discounted per person life expectancy (months) | 253.6 | 253.7 | 253.8 | 253.9 |
| Discounted per person costs ($) months | 370 | 373 | 381 | 409 |
| ***Cost-effectiveness ratio ($/YLS)*** | **---** | 700 | 900 | 1,900 |

|  | **HIV testing frequency** | | | |
| --- | --- | --- | --- | --- |
|  | **Current practice** | **One-time** | **Every 5 years** | **Annually** |
| **2x Base Case All Costs** |  |  |  |  |
| ***HIV-infected population*** |  |  |  |  |
| Discounted per person life expectancy (months) ^a^ | 184.2 | 188.5 | 196.1 | 208.3 |
| ***Overall population*** |  |  |  |  |
| Discounted per person life expectancy (months) | 253.6 | 253.7 | 253.8 | 253.9 |
| Discounted per person costs ($) months | 2206 | 2219 | 2251 | 2364 |
| ***Cost-effectiveness ratio ($/YLS)*** | **---** | 2,400 | 3,700 | 7,900 |
|  |  |  |  |  |
| **3 lines of ART (Base Case = 2 lines)** |  |  |  |  |
| ***HIV-infected population*** |  |  |  |  |
| Discounted per person life expectancy (months)^a^ | 185.5 | 190.5 | 199.0 | 212.7 |
| ***Overall population*** |  |  |  |  |
| Discounted per person life expectancy (months) | 253.6 | 253.7 | 253.8 | 254.0 |
| Discounted per person costs ($) months | 740 | 747 | 764 | 821 |
| ***Cost-effectiveness ratio ($/YLS)*** | **---** | 1,200 | 1,700 | 3,600 |

|  | **HIV testing frequency** | | | |
| --- | --- | --- | --- | --- |
|  | **Current practice** | **One-time** | **Every 5 years** | **Annually** |
| **Prior WHO treatment initiation criteria (ART initiation with CD4<250 or with WHO Stage III/IV OI)** |  |  |  |  |
| ***HIV-infected population*** |  |  |  |  |
| Discounted per person life expectancy (months)^a^ | 183.5 | 187.3 | 194.2 | 204.6 |
| ***Overall population*** |  |  |  |  |
| Discounted per person life expectancy (months) | 253.6 | 253.6 | 253.7 | 253.9 |
| Discounted per person costs ($) months | 738 | 744 | 760 | 814 |
| ***Cost-effectiveness ratio ($/YLS)*** | **---** | 1,300 | 2,100 | 4,200 |

PY – person-years; YLS – year of life saved

^a^Calculated from the time of model entry – includes time to HIV infection (incident cases only) and detection
