# Supplementary material for: Clinical Impact and Cost-Effectiveness of Expanded Voluntary HIV Testing in India
Source: PLoS One. 2013 May 31;8(5):e64604. doi: 10.1371/journal.pone.0064604 (PMC3669338; doi:10.1371/journal.pone.0064604)
Supplement: Table S2 — Sensitivity analysis on secondary transmission. 90% linkage-to-care. (DOCX) [file pone.0064604.s004.docx]

**Table S2. Sensitivity analysis on secondary transmission. 90% linkage-to-care.**

|  | | | | | |
| --- | --- | --- | --- | --- | --- |
|  | **HIV testing frequency** | | | | |
|  | **Current practice** | | **One-time** | **Every 5 years** | **Annually** |
| **National population** |  |  | |  |  |
| ***Prevalence 0.29%, Incidence 0.032/100PY, background testing 3.2% per year*** |  |  | |  |  |
| Number of secondary cases (per 100,000) | 6.93 | 6.60 | | 6.47 | 6.06 |
| Incremental HIV cases averted^a^ (per 100,000) | ---- | 0.33 | | 0.13 | 0.41 |
| % incremental decrease | ---- | 4.8 | | 2.0 | 6.3 |
|  |  |  | |  |  |
| **High prevalence district** |  |  | |  |  |
| ***Prevalence 0.8%, Incidence 0.088/100PY, background testing 3.3% per year*** |  |  | |  |  |
| Number of secondary cases (per 100,000) | 6.99 | 6.67 | | 6.52 | 6.15 |
| Incremental HIV cases averted^a^ (per 100,000) | ---- | 0.32 | | 0.15 | 0.37 |
| % incremental decrease | ---- | 4.6 | | 2.2 | 5.7 |
|  |  |  | |  |  |
| **High-risk group** |  |  | |  |  |
| ***Prevalence 5.0%, Incidence 0.552/100PY, background testing 50% per year*** |  |  | |  |  |
| Number of secondary cases (per 100,000) | 6.87 | 6.83 | | 6.80 | 6.67 |
| Incremental HIV cases averted^a^ (per 100,000) | ---- | 0.04 | | 0.03 | 0.13 |
| % incremental decrease | ---- | 0.6 | | 0.4 | 2.0 |
| PY – person-years  Transmission coefficient ranges from 0.16/100 PY to 9.03/100 PY depending on HIV RNA level. ([45](#_ENREF_45))  ^a^Incremental HIV cases averted relative to those averted with the next less frequent HIV testing strategy. | | | | | |
