# Supplementary material for: Clinical Impact and Cost-Effectiveness of Expanded Voluntary HIV Testing in India
Source: PLoS One. 2013 May 31;8(5):e64604. doi: 10.1371/journal.pone.0064604 (PMC3669338; doi:10.1371/journal.pone.0064604)
Supplement: Text S1 — Technical appendix. (DOCX) [file pone.0064604.s006.docx]

**Clinical impact and cost-effectiveness of expanded voluntary HIV testing in India**

**TECHNICAL APPENDIX**

Venkatesh, et al.

In areas where computer programming and methods are identical, the text from this appendix is similar to the online technical appendix available from:

Walensky R, Wood R, Fofana MO, Martinson NA, Losina E, et al. (2011) The clinical impact and cost-effectiveness of routine, voluntary HIV screening in South Africa. Journal of Acquired Immune Deficiency Syndrome 56:26-35.

Paltiel A, Weinstein M, Kimmel A, Seage Gr, Losina E, et al. (2005) Expanded screening for HIV in the United States--an analysis of cost-effectiveness. New England Journal of Medicine 352: 586-595.

**INTRODUCTION**

The analysis described in the main manuscript is based on the Cost-Effectiveness of Preventing AIDS Complications (CEPAC) International Model, a computer simulation of the clinical management and economics of HIV disease. The International Model employs data inputs from South Africa, India, and Côte d’Ivoire. The current analysis uses data specifically from India, when available. We have also linked this disease simulation to a model of HIV screening, detection, and referral-to-care. In addition to the details of the screening model provided in our prior work, the international screening model addresses issues critical to the international setting, including less complete access to care and sporadic case identification often associated with severe opportunistic diseases [[1](#_ENREF_1),[2](#_ENREF_2)]. In this Technical Appendix, we provide greater detail on the mechanics of both the CEPAC International Model and the Screening Model and provide results of additional sensitivity analyses performed in the context of this manuscript.

**METHODS**

The CEPAC International Model is a computer-based, state-transition, Monte Carlo simulation model of the progression and outcomes of HIV disease in a hypothetical cohort of patients in resource-limited settings. “State-transition” means that the model characterizes the natural history of illness in an individual patient as a sequence of monthly transitions from one “health state” to another. “Monte Carlo” refers to a random number generator and set of estimated probabilities that are used to determine the sequence of movements between health states for a particular patient. Each individual patient’s clinical course is followed from the time of entry into the model until death. A running tally is maintained of all clinical events, the length of time spent in each health state, and the costs and quality of life associated with each health state. Upon the patient’s death, summary statistics are recorded and a new patient enters the model. This process is then repeated over a large number of patients (statistical convergence can typically be achieved with cohort sizes of five to ten million), at which point, overall performance measures such as average life expectancy, quality-adjusted life expectancy, and cost are computed. They may be tallied independently for the Indian population in general and more specifically for those with HIV infection.

In the Disease Model, health states are chosen to be descriptive of the patient’s current health, relevant history, and resource utilization patterns. They are designed to be predictive of clinical prognosis, including disease progression, immune system deterioration, development and relapse of different opportunistic infections (OIs), toxic reactions to medications, resistance to therapy, and mortality. The model defines three general categories of health states: chronic, acute, and death. Most of the time, patients are in one of the chronic states, where progression of disease and immune system deterioration (i.e. CD4 decline) take place. Patients who develop an acute complication (i.e. an OI or drug-related toxicity) temporarily move to an acute health state, where both resource consumption levels and mortality rates are higher. Deaths can occur from either a chronic or an acute state and can be attributed to a particular OI, chronic AIDS (e.g., wasting), or non-AIDS-related causes.

The chronic and acute health states are stratified by: current and nadir CD4 cell count (>500 cells/µl; 301–500 cells/µl; 201–300 cells/µl; 101–200 cells/µl; 51–100 cells/µl; and 0-50 cells/µl) and current and set-point HIV RNA level (>30,000 copies/mL; 10,001–30,000 copies/ mL; 3,001–10,000 copies/ mL; 501–3,000 copies/ mL; 51-500 copies/ mL; 0-50 copies/mL). Drawing from distributions of patient characteristics (age, sex, CD4 count and HIV RNA level) derived from observational datasets, a patient is randomly assigned to a health state. By permitting the user to define initial population distributions for patient age, sex, CD4 cell count, HIV-RNA, and other demographic and clinical attributes, the model has the flexibility to explore a broad range of different patient cohorts.

At the start of each one-month cycle, the model records the patient’s CD4 count, HIV-RNA level, history of acute illness, and current therapies and uses these characteristics to determine the probabilities that indicate movement to a new state in the subsequent month. Monthly probabilities of events are estimated directly from published sources and available databases and are translated into risk functions for the model [[3](#_ENREF_3),[4](#_ENREF_4)].

These risk functions embody the key parameters of the natural history of HIV illness, AIDS, and OIs, including: rates of disease progression, OI risks, survival probabilities, and the effects of therapy. The model treats HIV RNA as the primary driver of immune system deterioration, and thus the assigned plasma viral load level determines the rate at which the patient’s CD4 count will decline in the absence of ART [[5](#_ENREF_5)]. Recognizing the absence of data suggesting that HIV RNA levels differ by geographic region or clade, we have validated that theUS-based data used for this purpose provide a reasonable surrogate for the dynamics between CD4 count and HIV RNA in resource-limited settings [[6](#_ENREF_6)]. Patients with a history of OIs have a higher risk of recurrence, depending on CD4 count and current use of ART [[3](#_ENREF_3)]. The model’s handling of efficacy and durability of ART are described elsewhere [[4](#_ENREF_4)]. Briefly, we estimate efficacy from data on viral suppression and CD4 count change over time, using observational data from India when available [[5](#_ENREF_5),[7](#_ENREF_7)]. From these data, we derive a probability of “early” (within six months) and “late” (beyond six months) failure for each first-line and second-line antiretroviral regimen to be considered. This structure allows patients to transition from virologic suppression to “failure” with the appropriate change in HIV RNA and CD4 count.

The clinical events within the model are predicated on model-generated “truth” regarding a patient’s CD4 count and viral load status. In resource-limited settings, however, this information may not be available to the provider due to limited access to laboratory monitoring. The CEPAC International Model retains the flexibility to make clinical decisions based on data available including: 1) clinical events alone; 2) CD4 count monitoring (with variable frequency); and/or 3) HIV RNA monitoring (with variable frequency). For the current analysis, each diagnosed HIV-infected patient is followed quarterly with clinic visits and bi-annually with CD4 counts, in accordance with current standards of HIV care in India [[8](#_ENREF_8),[9](#_ENREF_9)]. The model also has the flexibility to deviate from such standards to reflect incomplete clinic appointment or treatment adherence.

In addition to life expectancy and total lifetime costs, model outputs may include disaggregated estimates of average cost per patient (e.g., drug, laboratory, hospitalization), performance of therapy (e.g., time on therapy, time to virologic rebound) and morbidity (specific OI incidence rates, and causes of death). In general, most input data for the Disease Model have been described and published previously [[4](#_ENREF_4)].

**Screening Model**

The purpose of the Screening Model is to simulate the clinical impact of HIV counseling, testing and referral activities in a population and to convey this information to the Disease Model. The Disease Model combines this information with its own output on the timing of AIDS-defining illnesses (in the absence of treatment) to establish whether, when, and how an individual case of HIV infection is first detected. We use India-specific data to specify the parameters of relevance to the analysis, including country- and region-specific estimates of HIV prevalence and HIV test attributes, such as sensitivity, specificity, and cost [[10](#_ENREF_10),[11](#_ENREF_11),[12](#_ENREF_12),[13](#_ENREF_13),[14](#_ENREF_14)].

Individual members of the population enter the simulation, one at a time. A random-number generator makes use of “time-to-event” probability distributions to assign each incoming person a unique set of four, HIV-related event times: (1) time of HIV infection, t_i_; (2) time of next HIV test performed within the context of a specific HIV screening program to be evaluated, t_p_; (3) time of next HIV test performed within the context of any other “background” screening mechanism (such as an HIV test performed in an antenatal or tuberculosis clinic setting), t_b_; and (4) time of non-AIDS-related death, t_d_.

We treat the user-specified HIV incidence value as a hazard rate which, in the absence of reliable data, we assume to be constant over time. This rate, in turn, implies a monthly infection probability. Based on this probability, a random number generator within the model determines the individual patient’s time to infection. Similar methods are used to determine non-HIV-related mortality based on standard life tables [[4](#_ENREF_4)]. Instances where a patient’s time to non-HIV-related death exceeds the time to infection represent cases of actual infection during one’s lifetime.

In many instances, the assigned time to infection, t_i_, far exceeds the assigned time to death, t_d_ , reflecting the reality that many people die uninfected. A simple “IF/THEN” statement makes this determination; such cases will never enter the Disease Model. In a large fraction of cases, however, individuals are assigned values t_i_< t_d_ and will become HIV-infected during their lifetimes. At their given time t_i_, these patients proceed to the Disease Model, which transcribes their age at the moment of infection and proceeds to simulate the progress of their illness and its associated clinical and economic outcomes. However, these individuals are not eligible to receive any kind of HIV therapy within the Disease Model until and unless their HIV infection is identified. Thus, the Disease Model simulates the progress of disease for all infected individuals, but only detected cases are eligible for antiretroviral therapy and OI prophylaxis. Patients who are successfully detected and linked to care are assumed to receive services (and incur all concomitant costs) that conform to NACO guidelines.

Detection of HIV infection can occur via one of three discrete mechanisms: First, an infected individual can receive an HIV-positive test result within the context of a specific HIV screening program to be evaluated (In such a case, t_b_> t_p_> t_i_). Second, an infected individual can receive an HIV-positive test result within the context of any other background testing mechanism (hence, t_p_> t_b_> t_i_). Third, detection of HIV infection can take place because an infected individual seeks medical care for an AIDS-defining severe OI. The Screening Model determines the time of detection via the first two mechanisms, using a random number generator to combine user-specified assumptions regarding HIV test accuracy, program cost, and rates of test acceptance, and linkage-to-care, and background surveillance, as obtained from Indian data (see Table 1 in main text). This information is conveyed to the Disease Model which, in turn, determines the actual time of detection by comparing the time of screen-detection to the time of the first severe OI. To bias the results against a screening program, the model conservatively assumes that detection via either background testing or opportunistic disease development leads to perfect HIV test accuracy, acceptance, linkage-to-care and at no HIV testing cost. Routine HIV screening programs, in contrast, have “leakage” based on user-specified inputs on restrictions in test acceptance and linkage-to-care rates.

The Screening Model defines four, distinct HIV states: HIV-negative, HIV-acute, HIV-asymptomatic, and HIV-symptomatic. Patients newly infected with HIV are immediately moved from the HIV-negative state to the transient, HIV-acute state. Following their acute HIV infection, patients transition from the HIV-acute state to the chronic, HIV-asymptomatic state[[15](#_ENREF_15)]. The time for that transition is a user-specified option whose value in the current analysis is set to two months. Users can specify both pre- and post-seroconversion test sensitivities and specificities, thus capturing the “window” properties of alternative test protocols. Reflecting the rapid HIV test availability in India, we assume for the purpose of this analysis that the test specificity is zero during the “window” period.

Each individual’s experience in the Screening Model is tracked from the time of entry until either death or transition to the Disease Model. A running tally is maintained of all test offers and acceptances, success/failure to link to care, and all costs and quality-of-life effects associated with these events. Upon the patient’s departure from the Screening Model, summary statistics are recorded and a new patient simulation begins. Stable estimates of overall performance are obtained by aggregating large numbers (~ten million) of patient simulations. Sample size requirements depend upon user-specified assumptions of population prevalence, incidence, and testing program performance. Outputs of the screening model include: total program enrollment by HIV status; test acceptance; frequency of testing and total number and type of tests performed; CD4 cell count and HIV-RNA at the time of detection; time from HIV infection to diagnosis; mechanism of detection (program screening, background surveillance, or AIDS-defining illness); and total testing-related costs.

**CD4 Calibration**

The CD4 cell count at the time of ART initiation was also imputed with the CEPAC model using a cohort of acutely HIV-infected individuals (mean CD4 cell count of 553 cells/μl) with a background HIV testing rate of 3.2% per year and HIV detection with 10% of severe OIs and TB. This imputation yielded a mean CD4 cell count at ART initiation of 307 cells/μl, which was concordant with the CD4 cell count of HIV-infected individuals at the time of ART initiation at YRG CARE (305 cells/μl).

**HIV Prevalence and Incidence Estimates**

HIV prevalence values for India are taken from published data for each modeled population [[12](#_ENREF_12),[13](#_ENREF_13)]. Since HIV detection by screening necessarily applies only to those who have not previously been identified as HIV-positive, either because they are HIV-negative or undetected as HIV-positive, published population prevalence estimates are adjusted for the Screening Model to represent undetected HIV prevalence only. This undetected prevalence is calculated conservatively using published reports of the proportion of people in high-risk Indian populations who have been tested for HIV in the past twelve months and know their results [[16](#_ENREF_16)]. Prevalence inputs to the Screening Model are further adjusted for acute, undetected HIV cases and chronic, undetected HIV cases, using a two-month window of acute infection [[15](#_ENREF_15)].

Incidence inputs to the Screening Model in this analysis are calculated using the relationship: Prevalence = Incidence * Duration. Published population prevalence data are used for this calculation [[12](#_ENREF_12),[13](#_ENREF_13)]. The following prevalence data and computed incidence were used:

1) Indian general population (0.29% prevalence; 0.022/100 PY incidence); 2) high HIV prevalence districts (0.8% prevalence; 0.088/100 PY incidence); and 3) individuals identifying themselves in groups at high risk for HIV infection, specifically MSM, FSW, IDUs, and STD clinic attendees (5.0% prevalence, which is an estimated aggregate prevalence; 0.552/100 PY incidence). Duration of infection in India is calculated using life-expectancy outputs from the CEPAC International Disease Model. A cross-sectional population is modeled using several runs of the Disease Model with separate cohorts, including a cohort without ART and cohorts with varying immunologic or clinical ART initiation criteria. The life expectancy results from the time of model initiation for these runs are then weighted into an average duration of HIV infection in the population. The weights are determined by published data on the percentage of the HIV-infected Indian population receiving ART and an estimated distribution of immunologic initiation criteria for those receiving ART [[16](#_ENREF_16)].

**Estimating Secondary Infections**

The success of HIV testing, ART, and behavioral counseling in reducing transmission of HIV infection can be gauged by the reductions in secondary infections and increases in life expectancy they produce. In order to estimate the number of secondary cases in the “current practice” scenario and each HIV screening scenario, we first determine the total number of person-months spent in each HIV RNA stratum, starting from each patient’s entry into the model. These values are multiplied by probabilities of transmission by HIV RNA level, derived from published international transmission rates [[17](#_ENREF_17)], to find the total number of secondary infections in each HIV RNA stratum. To determine the total number of secondary infections in the cohort, we add the secondary infections from all HIV RNA levels.

Given the uncertainty surrounding the effect of ART on secondary transmissions, we consider a range of plausible scenarios, including increasing test acceptance to 90% as well as increasing linkage-to-care to 90%.

**RESULTS**

Results reported in the text provide detailed information regarding sensitivity analyses on the most influential input parameters. We first present sensitivity analyses for HIV testing among the national population comparing current practice to one-time, every 5 years, and annual HIV screening (Table S1). We then present tornado diagrams for sensitivity analyses for HIV testing among high prevalence districts and high-risk groups comparing one-time to every 5 year HIV screening (Figures S1 and S2).

The following parameters are varied in the sensitivity analyses presented below: increasing and decreasing HIV prevalence by 50%, background testing at 10% per year, varying HIV test acceptance from 30% to 90%, varying linkage-to-care from 20% to 90%, varying the base case test cost from 0.5 times to 2 times, halving the base case cost of second-line ART only, halving the base case cost of both first- and second-line ART, varying the base case for all costs from 0.5 times to 2 times, providing three lines of ART, and utilizing the prior WHO treatment initiation criteria of starting ART with a CD4 count< 250 cells/ul or with a WHO stage III or IV OI.

In the case of the secondary transmission analyses, we also present the number of cases of HIV infection prevented when increasing linkage-to-care to 90% (Table S2a), as well as when increasing the test acceptance to 90% (Table S2b) in the national population.

**REFERENCES**

1. Paltiel A, Walensky R, Schackman B, Seage G, Mercincavage L, et al. (2006) Expanded HIV screening in the United States: effect on clinical outcomes, HIV transmission, and costs. Annals of Internal Medicine 145: 797-806.

2. Paltiel A, Weinstein M, Kimmel A, Seage G, Losina E, et al. (2005) Expanded screening for HIV in the United States--an analysis of cost-effectiveness. N Engl J Med 352: 586-595.

3. Holmes C, Wood R, Badri M, Zilber S, Wang B, et al. (2006) CD4 decline and incidence of opportunistic infections in Cape Town, South Africa: implications for prophylaxis and treatment. Journal of Acquired Immune Deficiency Syndrome 42: 464-469.

4. Walensky R, Wood R, Weinstein M, Martinson N, Losina E, et al. (2008) Scaling up antiretroviral therapy in South Africa: the impact of speed on survival. J Infect Dis 197: 1324-1332.

5. Mellors J, Muñoz A, Giorgi J, Margolick J, Tassoni C, et al. (1997) Plasma viral load and CD4+ lymphocytes as prognostic markers of HIV-1 infection. Ann Intern Med 126: 946-954.

6. Goldie S, Yazdanpanah Y, Losina E, Weinstein M, Anglaret X, et al. (2006) Cost-effectiveness of HIV treatment in resource-poor settings--the case of Côte d'Ivoire. N Engl J Med 355: 1141-1153.

7. Cecelia A, Christybai P, Anand S, Jayakumar K, Gurunathan T, et al. (2006) Usefulness of an observational database to assess antiretroviral treatment trends in India. Natl Med J India 19: 14-17.

8. National AIDS Control Organisation (2007) Antiretroviral Therapy guidelines for adults and adolescents including post-exposure prophylaxis New Delhi: Ministry of Health and Family Welfare, Government of India.

9. National AIDS Control Organisation (2007) Guidelines for Prevention and Management of Common Opportunistic Infections/Malignancies among HIV-Infected Adults and Adolescents. New Delhi: Ministry of Health and Family Welfare, Government of India.

10. Bhore A, Sastry J, Patke D, Gupte N, Bulakh P, et al. (2003) Sensitivity and specificity of rapid HIV testing of pregnant women in India. Int J STD AIDS 14: 37-41.

11. Dandona L, Kumar S, Ramesh Y, Rao M, Kumar A, et al. (2008) Changing cost of HIV interventions in the context of scaling-up in India. AIDS 22: S43-49.

12. National AIDS Control Organisation (2010) Annual Report 2010-2011. Ministry of Health and Family Welfare.

13. National AIDS Control Organisation (2006) Technical Report on HIV Estimation, 2006. Ministry of Health and Family Welfare.

14. International Institute for Population Sciences (2007) National Family Health Survey (NFHS-3), 2005-2006.

15. Walensky R, Goldie S, Sax P, Weinstein M, Paltiel A, et al. (2002) Treatment for primary HIV infection: projecting outcomes of immediate, interrupted, or delayed therapy. J Acquir Immune Defic Syndr 31: 27-37.

16. UNAIDS (2010) Country Progress Report UNGASS India.

17. Attia S, Egger M, Müller M, Zwahlen M, Low N (2009) Sexual transmission of HIV according to viral load and antiretroviral therapy: systematic review and meta-analysis. AIDS 23: 1397-1404.
